# Lipocalin-2 modulates recipients alloimmune responses to the murine kidney transplants

**DOI:** 10.3389/fimmu.2025.1716393

**Published:** 2025-12-19

**Authors:** Anna Maria Pfefferkorn, Raphaela Fritsche-Guenther, Angelika Kusch, Hubert Schwelberger, Shiqian Liu, Robert Klopfleisch, Yuhuan Li, Rusan Catar, Shaokun Liu, Felix Aigner, Johann Pratschke, Igor Maximilian Sauer, Muhammad Imtiaz Ashraf

**Affiliations:** 1 Department of Surgery, Experimental Surgery, Charité – Universitätsmedizin Berlin, Corporate Member of Freie Universität Berlin, Humboldt-Universität zu Berlin and Berlin Institute of Health, Berlin, Germany; 2 Berlin Institute of Health at Charité – Universitätsmedizin Berlin, Metabolomics Platform, Berlin, Germany; 3 Department of Nephrology and Internal Intensive Care Medicine, Charité – Universitätsmedizin Berlin, Corporate Member of Freie Universität Berlin, Humboldt-Universität zu Berlin and Berlin Institute of Health, Berlin, Germany; 4 Department of Visceral, Transplant and Thoracic Surgery, Medical University, Innsbruck, Austria; 5 Institiute of Pathology, Department of Veterinary Medicine, Freie Universität Berlin, Berlin, Germany; 6 Translational Radiation Oncology Research Laboratory, Department of Radiooncology and Radiotherapy, Charité - Universitätsmedizin Berlin, Corporate Member of Freie Universität Berlin, Humboldt-Universität zu Berlin and Berlin Institute of Health, Berlin, Germany; 7 Department of Surgery, Krankenhaus der Barmherzigen Brüder, Graz, Austria

**Keywords:** allograft rejection, alloimmune responses, ischemia-reperfusion injury, lipocalin-2, mouse kidney transplantation

## Abstract

**Background:**

Lipocalin-2 (Lcn2) is a sensitive early marker for acute kidney injury, delayed graft function and acute rejection of kidney transplants. We previously showed the renoprotective effect of recombinant Lcn2:Siderophore: Fe3^+^ (rLcn2) in a mouse kidney transplantation (KTx) model. Here, we investigate the molecular and cellular mechanisms underlying these effects.

**Methods:**

Male C57BL/6 mice (10–12 weeks) received BALB/c kidney allografts, with or without rLcn2 treatment (250 µg, s.c.). To examine the immunomodulatory function of rLcn2, immune cells from graft, spleen, lymph nodes and blood were analyzed by flow cytometry at post-operative days (pod) 3 and 7. Syngeneic C57BL/6 grafts were used to investigate the impact of rLcn2 on alloimmune-independent tissue injury and inflammation through multiplex signaling assays, functional readouts, cytokine profiling and histopathological analyses.

**Results:**

rLcn2 treatment markedly reduced frequencies of distinct T cell subsets, including effector memory T cells and their cytotoxic (T_c_) and helper (T_h_) subsets across grafts, lymphoid tissues and blood by pod-7 following allogeneic KTx. In graft infiltrating CD8^+^ T cells, rLcn2 decreased degranulation capacity and diminished expression of interferon-γ and perforin. rLcn2 also lowered the proportion of NKG2D^+^ CD8^+^ T cells, an activating T_c_ subset, in spleen and blood. In contrast, its impact on innate immune cells was modest and selective, influencing only neutrophils, macrophages in lymph nodes and intermediate mature NK cells in spleen and blood. No significant effect of rLcn2 treatment was observed on alloimmune-independent tissue injury or inflammation in syngeneic kidney grafts.

**Conclusion:**

rLcn2 selectively modulates T-cell activity after KTx without affecting alloimmune-independent injury pathways.

## Introduction

Acute rejection is a significant challenge in kidney transplantation, representing a critical obstacle to the long-term success of allografts (1–3). Despite advancements in immunosuppressive therapies and transplant protocols, the immune system’s ability to recognize and mount responses against the transplanted organ remains a significant concern. This immune-mediated injury, comprising cellular and antibody-mediated rejection, jeopardizes graft survival and patient health (4). The complexities of acute rejection lie in its intricate immunological mechanisms, involving a cascade of events triggered by allorecognition and subsequent immune activation. The interplay of various immune cells, cytokines, and signaling pathways orchestrates the rejection process, culminating in tissue damage and functional impairment of the transplanted kidney. The timely diagnosis and management of acute rejection episodes are crucial to preventing irreversible damage to the allograft and preserving long-term graft function (5, 6). Efforts to prevent, detect, and treat acute rejection are pivotal focal points in kidney transplantation research, aiming to improve outcomes and enhance the longevity of transplanted kidneys.

Lipocalin-2 (Lcn2), also known as neutrophil gelatinase-associated lipocalin (NGAL), is a glycoprotein primarily secreted by neutrophils and plays a crucial role in antibacterial innate immune responses through iron-siderophore binding (7–10). Moreover, Lcn2 regulates immune responses by stimulating chemotaxis and influencing cell growth, differentiation, and apoptosis through modulation of iron homeostasis (11–14). The levels of Lcn2 in blood serum and urine serve as potential biomarkers for acute kidney injury (AKI) and renal graft rejection (15–17). However, the functional role of Lcn2 in AKI remains debated. Some studies suggest protective properties of Lcn2 (18–20), while others propose detrimental effects such as excessive autophagy, tissue damage, and cell death (21). While the presence of Lcn2 in allograft biopsies correlates with the severity of AKI post-transplantation (22), there is limited data on the biological functions and metabolism of endogenously synthesized Lcn2 in kidney allografts, as well as the effects of exogenously administered recombinant Lcn2:Siderophore: Fe complex (rLcn2) in recipients.

In the context of kidney transplantation, prior investigations utilizing a mouse model have shown that, unlike endogenously produced Lcn2, the perioperative administration of recombinant Lcn2:siderophore:Fe complex (rLcn2) significantly reduces allograft damage and enhances graft function during the early phases of rejection (23). These findings highlight the potential of rLcn2 as a renoprotective agent, though the precise underlying mechanisms of this protective effect remain unclear. To evaluate how rLcn2 confers graft protection, we aimed to simultaneously examine the two early challenges that transplants face: the recipient’s alloimmune response to donor mismatch and alloimmune-independent ischemia–reperfusion injury (IRI). To this end, we performed detailed phenotypic and functional analysis of adaptive and innate immune cells, primarily T and NK cell subsets from the graft, spleen, lymph nodes and blood after allogeneic kidney transplantation (BALB/c-C57BL/6). In parallel, we assessed stress, inflammatory and survival signaling in the syngeneic grafts (C57BL/6-C57BL/6). Our results suggest that rLcn2 exerts its protective effects on allografts primarily by modulating CD8^+^ T cell-mediated alloimmune responses rather than alloimmune independent, IRI-related mechanisms.

## Materials and methods

### Animals

Male C57BL/6 and BALB/c mice, aged (10–12 weeks) were used for all the experiments. The animals were purchased from Janvier (Le Genest St Isle, France) and housed under standard conditions, with *ad libitum* access to food and water. All animal procedures were performed in accordance with the directive 2010/63/EU, the German Tierschutz-Versuchstierverordnung, and were approved by the Regional Ethics Committee for Animal Research (Landesamt für Gesundheit und Soziales Berlin, approval number: G0236/18).

### Mouse kidney transplantation

Mouse kidney transplantation was carried out under isoflurane anesthesia in both allogeneic (BALB/c to C57BL/6; n = 7-8) and syngeneic (C57BL/6 to C57BL/6; n = 5) strain combinations, as described previously (23). In short, after a midline abdominal incision, the donor’s left kidney, aorta and inferior vena cava were carefully dissected. The kidney was flushed *in situ* with histidine-tryptophane-ketoglutarate (HTK) solution (Custodiol^®^, Dr. Franz Köhler Chemie GmbH, Bensheim, Germany) and then excised *en bloc* with the renal vessels and ureter. The graft was stored in cold HTK solution until implantation into the nephrectomized recipient. Vascular anastomoses to the recipient’s abdominal aorta and vena cava were done using a knotless suture technique (24), and the ureter was connected directly to the bladder. In the rLcn2 treatment group, donor kidneys were flushed with HTK solution containing 250 µg of rLcn2, and recipients received a subcutaneous injection of 250 µg of rLcn2 15 minutes prior to graft reperfusion. Control animals received an equal volume of phosphate buffered saline (PBS). The concentration of rLcn2 was adapted from our previous study (23), which was initially determined and validated through preliminary titration experiments. Animals which experienced intraoperative or early postoperative death, or those in which technical complications such as vascular or ureteral issues were identified during the final graft assessment, were excluded from the study.

### Immune cell isolation

Immune cells were systematically isolated from kidney grafts, spleens, lymph nodes, and blood samples of allogeneically transplanted mice at either pod-3 or pod-7.

#### Spleen

The spleens were mechanically dissociated by cutting them into smaller pieces and gently grinding them through a 100 µm cell strainer using the syringe plunger and continuously rinsing with mouse medium (RPMI-1640, supplemented with L-Glutamine, 10% heat-inactivated fetal bovine serum [FBS] and 100 µg/mL Penicillin/Streptomycin). The resulting cell suspension was centrifuged at 335 g for 10 minutes at 4°C. After discarding the supernatant, erythrocytes were lysed by incubating the cell pellet with 5 mL of ACK lysis buffer (NH_4_Cl, KHCO_3_, Na_2_EDTA, pH 7.2) for 4 minutes. The cells were then washed with PBS containing 1% BSA (PBS + 1% BSA) and counted using a CASY counter (OMNI Life Science GmbH & Co KG, Bremen, Germany).

#### Kidney graft

Murine kidney grafts were minced into small pieces and digested in 6.0 mL of mouse medium containing 1 mg/mL collagenase IV (Gibco/Invitrogen, Darmstadt, Germany) and 200 U/mL DNase (Ambion/Applied Biosystems, Darmstadt, Germany), while shaking in a water bath at 37°C for 45 minutes. The enzymatic reaction was stopped by adding ice-cold mouse medium, followed by resuspension of the tissue for 3 minutes. The tissue suspension was filtered through a 100 µm cell strainer using a syringe plunger and rinsed with 25 mL of ice-cold mouse medium. The filtrate was centrifuged at 80 g for 8 minutes at 4°C to pellet residual tissue debris. The supernatant was transferred to a new tube and centrifuged at 335 g for 10 minutes at 4°C. The final cell pellet was resuspended in PBS + 1% BSA and leukocytes were enriched using CD45 MicroBeads (Miltenyi Biotec B.V. & Co, Bergisch Gladbach, Germany) according to the manufacturer’s instructions. Cell counts were determined using a CASY counter.

#### Lymph nodes

The mouse lymph nodes were carefully harvested from the mesentery of the recipient mice. Then, they were gently dissociated by passing through a 100 µm cell strainer using the syringe plunger in the presence of mouse medium. The resulting cell suspension was centrifuged at 335 g for 10 minutes at 4°C. The cell pellet was resuspended in PBS + 1% BSA and the cell was determined by the CASY counter.

#### Blood

Roughly 1 mL of blood was collected from the inferior vena cava of mice and centrifuged (2,000 g, 10 min, 4°C) to separate the serum. The serum was subsequently used for creatinine and urea analysis. The residual blood cell pellet was resuspended in 1 mL of PBS + 1% BSA. Then 700 µL of the suspension was transferred to a 50 mL Falcon**^®^** tube containing 7 mL of ACK lysis buffer. The mixture was then gently inverted 10 times and placed on a roll mixer for 2 minutes to facilitate erythrocyte lysis. Following centrifugation (400 g, 5 minutes, 4°C), the supernatant was decanted. The cell pellet was then resuspended in 10 mL of fresh ACK lysis buffer, gently inverted 5 times and incubated for 2 minutes at room temperature. The lysis reaction was stopped by adding 10 mL of PBS + 1% BSA. Subsequently, the cells were centrifuged at 400 g for 5 minutes at 4°C. After discarding the supernatant, the cell pellet was resuspended in 3 mL of PBS + 1% BSA and the total cell number was determined using a CASY counter.

### Flow cytometry

For flow cytometric immunophenotyping, approximately 1x10^6^ cells were first incubated for 10 minutes at 4°C in PBS containing 10% FcR Blocking reagent (Miltenyi Biotec B.V. & Co, Bergisch Gladbach, Germany). Then, the cells we incubated for 20 minutes at 4°C with fluorochrome-conjugated antibodies, distributed across four cytometry panels: NK-panel, T-panel, Innate-panel, and Functional-panel (**Supplementary Table S1**). Prior to use, the working concentrations of all antibodies were optimized, ensuring an ideal equilibrium between signal intensity for positive and negative populations. Relative immune cell frequencies were determined by acquiring 50,000 - 100,000 events in a live gate on a BD LSR Fortessa Cell Analyzer (BD Biosciences, San Jose, California, USA) and analyzing the data with FlowJo™ software v10.0 (Tree Star Inc., Ashland, OR, USA). For t-distributed stochastic neighbor embedding (t-SNE) analysis, concatenated FCS files containing 50,000 lymphocyte events each were processed using FlowJo™ default parameters (1,000 iterations, perplexity 30).

### Functional analysis of lymphocytes

To perform functional analysis of the lymphocytes, the immune cells were isolated from kidney grafts and spleens of the recipient mice and rested overnight in mouse medium, supplemented with 200 U/mL of mouse IL-2 (Miltenyi Biotec B.V. & Co, Bergisch Gladbach, Germany), at 37°C with 5% CO_2_. The following day, cells were stimulated for 4 hours at 37°C and 5% CO_2,_ using a 2 µL/mL of cell stimulation cocktail (eBioscience™ | Thermo Fisher Scientific Inc., Waltham, Massachusetts, USA) in the presence of 200 U/mL mouse IL-2, 5 µg/mL brefeldin A (Cayman Chemical, Ann Arbor, Michigan, USA), 2 µM of monensin (BioLegend, San Diego, California, USA), and anti-CD107a (LAMP-1) antibody.

After stimulation, the cells were stained with surface markers, fixed and permeabilized using the Foxp3/Transcription Factor Staining Buffer Set (eBioscience™ | Thermo Fisher Scientific Inc., Waltham, Massachusetts, USA) according to the manufacturer’s instructions. Then the cells underwent intracellular staining for 30 minutes at 4°C. Data acquisition was performed on an LSR Fortessa and analysis was conducted using FlowJo™ software.

### *In vitro* T cell activation

Immune cells were isolated from euthanized naïve C57BL/6 mice (n = 6 per experiment) and enriched for T cells using the Pan T Cell Isolation Kit II (Miltenyi) following the manufacturer’s instructions. Purified T cells were then seeded in 12-well flat-bottom plates at a density of 2 x10^6^ cells/well in mouse medium supplemented with IL-2 (200 U/mL). The cells were then stimulated using the ImmunoCultTM Mouse T Cell Activator kit (STEMCELL) following the manufacturer’s protocol, in the presence or absence of rLcn2 (2 µg/mL) for 72 hours. Thereafter, cells were harvested and stained with a panel of antibodies including canonical T cell markers (CD3, CD4, CD8) and NKG2D, followed by flow cytometric analysis.

### Multiplex immunoassay

Kidney tissue samples were obtained from syngeneically transplanted C57BL/6 wild-type mice (n = 5 per group), promptly frozen in liquid nitrogen, and stored at -80°C until further processing. Tissue lysates were prepared in 750 µL of cold RIPA buffer (1% NP-40, 1% CHAPS, 0.1% SDS, 0.15 mM NaCl, 10 mM Na-Phosphate, 2 mM EDTA, 50 mM NaF) supplemented with 1 mM complete protease inhibitor cocktail (Roche Diagnostics, Basel, Switzerland) and 0.2 mM Na-orthovanadate. Homogenization was performed using a Mixer Mill (MM400, Retsch GmbH, Haan, Germany) at a frequency of 30.0 Hz for 2 min. Protein concentrations were determined with the Pierce™ BCA Protein Assay Kit (Thermo Fisher Scientific Inc., Waltham, MA, USA) according to the manufacturer’s instructions.

Multiplex analysis was performed using the MILLIPLEX^®^ Multi-Pathway 9-Plex Magnetic Bead Kit and the MILLIPLEX^®^ Akt/mTOR Phosphoprotein Magnetic Bead 11-Plex Kit (Merck KGaA, Darmstadt, Germany). For each sample, 10 µg of total protein was added to individual wells of a V-shaped 96-well plate in a randomized order. β-actin was included as an internal control. Five quality control, samples comprising pooled proteins from all test samples were included on each plate to assess assay performance. All steps were performed according to the manufacturer’s protocol. Bead-based assays were read using the MagPix^®^ system (Luminex Corporation, Austin, TX, USA).

### Graft function

The function of the mouse renal grafts was assessed by measuring serum levels of creatinine and urea using specific enzymatic assays on a Roche Cobas analyzer as previously described (25).

### Histology

Mouse kidney graft samples were fixed in 4% paraformaldehyde for 24 hours, followed by transfer into 330 mOsmol/L sucrose in PBS containing 0.02% sodium azide, until further histological processing and paraffin embedding. Paraffin-embedded tissues were sectioned at 2-3 μm thickness, deparaffinized, and stained with hematoxylin and eosin (H&E). Histological evaluations were performed by a pathologist, blinded to the experimental groups (n = 5 per group). Acute tubular injury was assessed by scoring the percentage of tubules showing atrophy, loss of brush borders, cast formation, and dilatation as follows: 0 (none), 1 (<25%), 2 (26–50%), 3 (51–75%), and 4 (>75%). For each kidney, approximately 10 high-power fields (HPFs; 400× magnification) were analyzed.

### RNA isolation and cDNA synthesis

Total RNA was extracted from the mouse kidney grafts using the RNeasy Mini Kit (Qiagen, Hilden, Germany) with RNase-free DNase treatment following the manufacturer’s protocols. RNA quantity and quality were assessed using a NanoDrop Spectrophotometer (Thermo Scientific, Waltham, MA, USA). For cDNA synthesis, the High-capacity cDNA Reverse Transcription Kit (Qiagen, Hilden, Germany) and 2 µg of total RNA were employed following the manufacturer’s instructions.

### Quantitative real time-PCR

The qRT-PCR analysis was conducted using 1 µL of synthesized cDNA and TaqMan™ Fast Advanced Master Mix (Qiagen, Hilden, Germany), following the manufacturer’s instructions. The relative expression levels of target mRNAs were normalized to the β-actin (Actb) as the endogenous control. The calculations were carried out utilizing the 2-^ΔΔ^Ct method. Details of the mRNA expression assays are provided in **Supplementary Table S2**.

### Preparation of recombinant Lcn2

Mouse recombinant Lcn2 (rLcn2) was prepared as previously described (26). Briefly, Lcn2 lacking the signal peptide sequence was expressed in *E. coli* BL21 as a GST fusion protein that was purified by chromatography on Glutathione Sepharose (GSTrap FF, GE Healthcare, Vienna, Austria) and cleaved with thrombin protease. Mouse rLcn2 was purified to greater than 99% purity by chromatography on CIM-SO3 (BIA Separations, Ljubljana, Slovenia). To generate the Lcn2:Siderophore: Fe³^+^ complex, the purified rLcn2 was incubated with an equimolar amount of ferric enterobactin (EMC microcollections, Tübingen, Germany) for 4 hours at 4°C and subsequently dissolved in PBS at 1 mg/mL. This complex is highly stable at neutral pH, as previously shown for human Lcn2, and dissociates only under acidic or denaturing conditions (27). Given the high sequence and structural homology between human and mouse Lcn2, similar stability is expected for mouse rLcn2. rLcn2 was quantified by a commercial Bradford assay based on Coomassie Brilliant Blue G-250 dye binding (Bio-Rad Protein Assay, Bio-Rad Laboratories, Vienna, Austria) that had been calibrated with purified rLcn2.

### Statistical analyses

Statistical analyses were conducted using GraphPad Prism 9. For the comparison of two groups, the Mann-Whitney U Test was used, whereas for multiple comparisons involving normally distributed data, an ordinary one-way ANOVA was employed, alongside a Kruskal-Wallis test with Dunn’s *post-hoc* test for non-normally distributed data. Additionally, variations between groups relative to variations within groups were assessed using two-way ANOVA. Unless specified otherwise, data are expressed as mean values ± standard deviation (SD). Calculations resulting in a p-value of ≤ 0.05 were deemed statistically significant.

## Results

### rLcn2 selectively modulates T cell subsets after kidney transplantation

**Figure 1:**
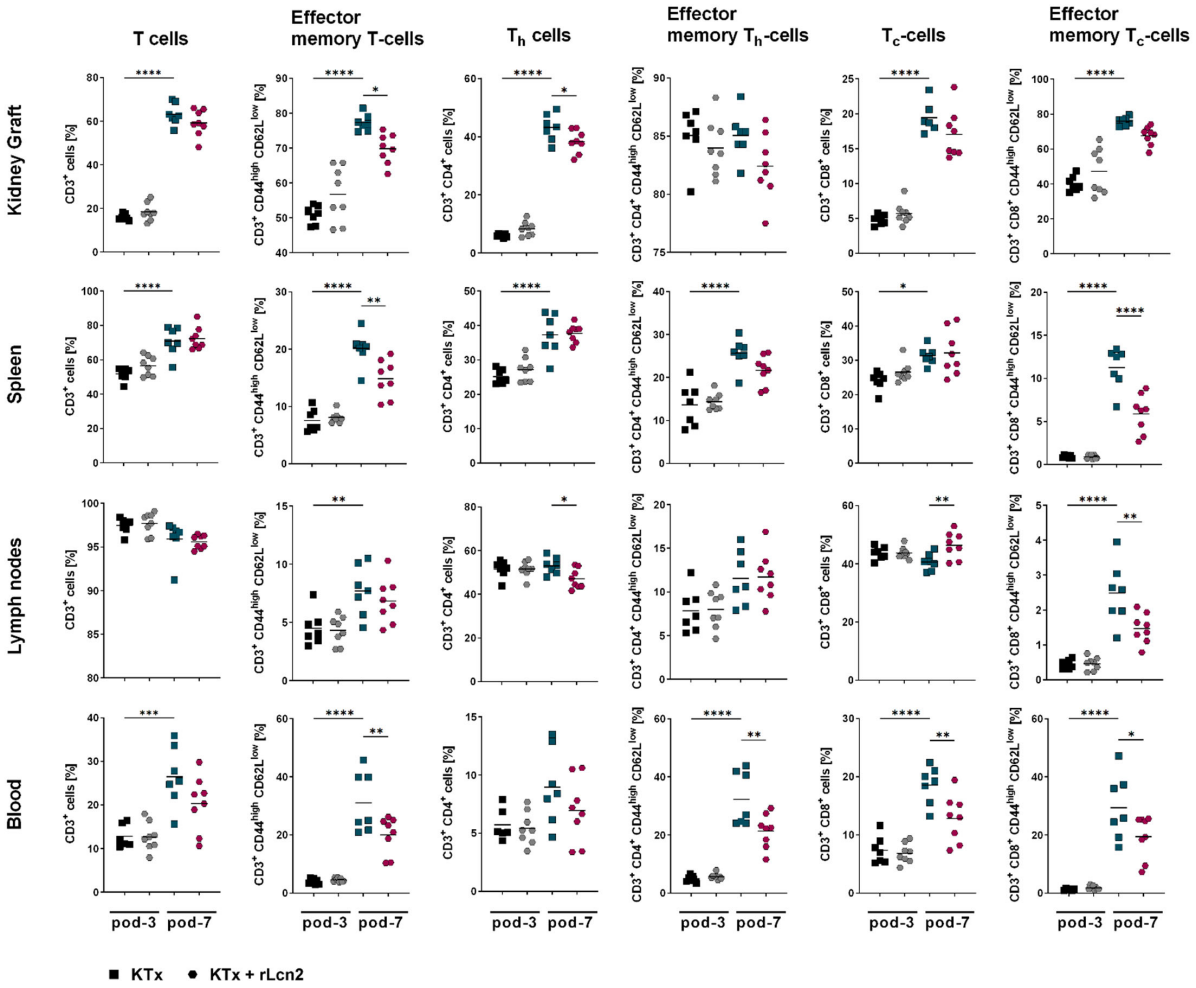
rLcn2 suppresses effector T cells after kidney transplantation. BALB/c to C57BL/6 kidney transplant recipients received perioperative rLcn2 (250 µg, s.c.) or vehicle. T cells, including helper (T_h_) and cytotoxic (T_c_) subsets and their effector memory populations, were analyzed in kidney grafts, spleens, lymph nodes, and blood at pod-3 and pod-7 by flow cytometry. Gating strategies are described in **Supplementary Figure S1**. rLcn2 treatment reduced effector memory T cells, T_h_, and T_c_ subsets at pod-7 across multiple tissues, while total T cell numbers remained unchanged. Scatter plots show the relative frequencies (%) of the indicated cell types. Data are presented as mean (n = 7 KTx pod-3 and pod-7, n = 8 KTx + rLcn2 pod-3 and pod-7); statistical significance was determined by ordinary one-way ANOVA (*p < 0.05, **p ≤ 0.01, ***p ≤ 0.001, ****p ≤ 0.0001).

rLcn2 has been shown to ameliorate kidney function and histological lesions associated with acute cellular rejection (ACR) following mouse kidney transplantation (23). To elucidate whether rLcn2 achieves that by modulating T cell-mediated alloimmune response, we phenotyped immune cells isolated from murine kidney grafts, secondary lymphoid tissues (spleen and lymph nodes) and blood at pod-3 and pod-7 following allogeneic kidney transplantation (BALB/c-C57BL/6) with or without perioperative rLcn2 treatment. Overall, T cell and their subset frequencies were higher at pod-7 compared to pod-3 (**Figure 1**). While the pan T cell number remained unchanged between untreated and rLcn2-treated recipients, a significant decrease in the frequencies of effector memory subset of T cells was observed in kidney grafts, spleens and blood samples of rLcn2-treated mice at pod-7. Likewise, rLcn2 treatment significantly reduced the frequencies of T_h_ cells in kidney grafts and lymph nodes, as well as effector memory T_h_ cells in blood at pod-7. Additionally, significant reductions in T_c_ cell frequencies in blood and effector memory T_c_ cells in spleens, lymph nodes and blood were observed in the rLcn2-treated mice at pod-7. In summary, perioperative treatment with rLcn2 distinctly modulates T cells and their subsets in kidney grafts, spleens, lymph nodes, and blood at pod-7, indicating its targeted immunomodulatory effects in mitigating alloimmune responses.

### rLcn2 selectively attenuates effector functions of T lymphocytes

**Figure 2:**
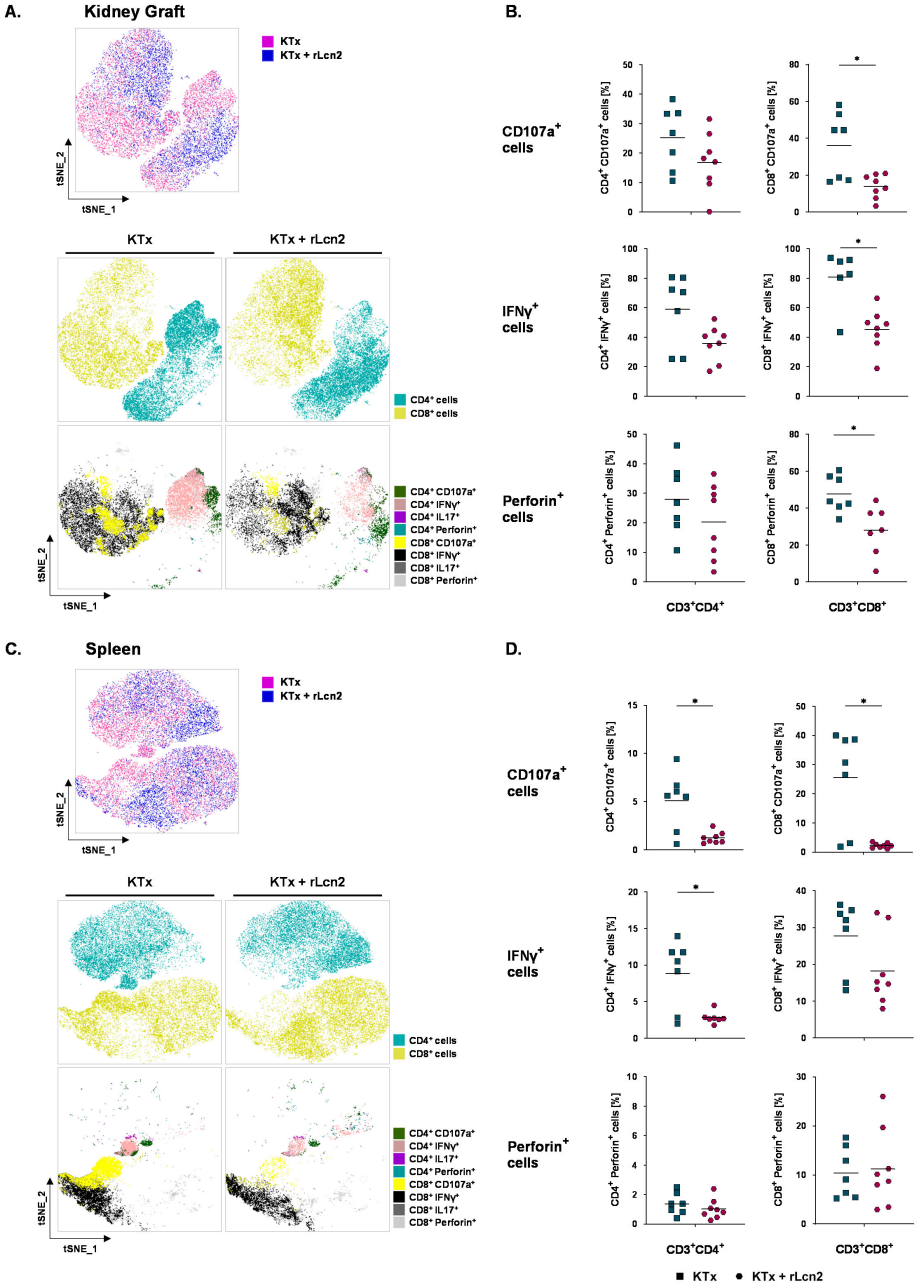
rLcn2 modulates T-cell function after kidney transplantation. BALB/c to C57BL/6 kidney transplant recipients received perioperative rLcn2 (250 µg, s.c.) or vehicle. Immune cells were isolated from kidney grafts and spleens at pod-7 and analyzed by flow cytometry for IFNγ, CD107a, and perforin expression following stimulation with PMA/Ionomycin. **(A, C)** Visualization of t-distributed stochastic neighbor embedding (t-SNE) analysis, depicting CD4^+^ and CD8^+^ T cells, as well as subsets expressing CD107a, IFNγ, IL-17, or perforin in kidney grafts **(A)** and spleens **(C)**, comparing rLcn2-treated versus untreated controls. t-SNE plots were generated by concatenating FCS files and utilizing 50,000 lymphocyte events per file. Default FlowJo™ parameters were applied (1000 iterations, perplexity of 30). Each data point represents an individual cell, and color-coding indicates distinct cell clusters. **(B, D)** Scatter plots demonstrate comparison of relatively frequencies (%) of the manually gated CD107a, IFNγ or Perforin positive CD4^+^ and CD8^+^ T cells in kidney grafts **(B)** and spleen **(D)** between rLcn2 treated and untreated groups. Gating strategies are shown in **Supplementary Figure S2**. rLcn2 reduced degranulation (determined by CD107a expression) and expression of IFNγ and perforin in graft-infiltrating CD8^+^ T cells and decreased degranulation in splenic T cell subsets, with minimal effects on other functions. Data are presented as mean (n = 7 KTx, n = 8 KTx + rLcn2); statistical significance was determined by ordinary one-way ANOVA (*p < 0.05).

The extent of allograft damage is primarily determined by the activity and cytotoxic potential of graft-infiltrating T lymphocytes, which drive tissue damage through the release of pro-inflammatory cytokines, cytolytic granules, and other effector molecules. To examine whether rLcn2 treatment modulates functionality of these lymphocytes, immune cells were isolated from kidney grafts of rLcn2-treated and untreated recipients at pod-7 and the functional activities of both CD4^+^ and CD8^+^ T cells were assessed by measuring cytokine production (interferon-γ, IFNγ), degranulation capacity (CD107a expression), and perforin expression. While functional activities of intragraft CD4^+^ T cells remained unchanged between rLcn2-treated and untreated mice, CD8^+^ T cells isolated from kidney grafts of rLcn2-treated demonstrated a significant reduction in both degranulation capacity and the ability to secrete IFNγ and perforin compared to untreated mice (**Figures 2A, B**). Moreover, to examine the effect of rLcn2 on systemic alloimmune activity, the functional analysis of the lymphocytes isolated from one of the secondary lymphoid organs, i.e. spleens of rLcn2-treated and untreated recipients was performed. Interestingly, both CD4^+^ and CD8^+^ T cell subsets isolated from spleens of rLcn2 treated mice exhibited a significant reduction in their degranulation capacities compared to untreated controls (**Figures 2C, D**). Similarly, CD4^+^ T cells from the spleens of treated mice showed a marked decrease in their potential to secrete IFNγ. However, rLcn2 treatment did not influence IFNγ production in splenic CD8^+^ T cells, nor did it influence perforin production in both T cell subsets.

### rLcn2 preferentially modulates NKG2D^+^ CD8^+^ T cells after kidney transplantation

**Figure 3:**
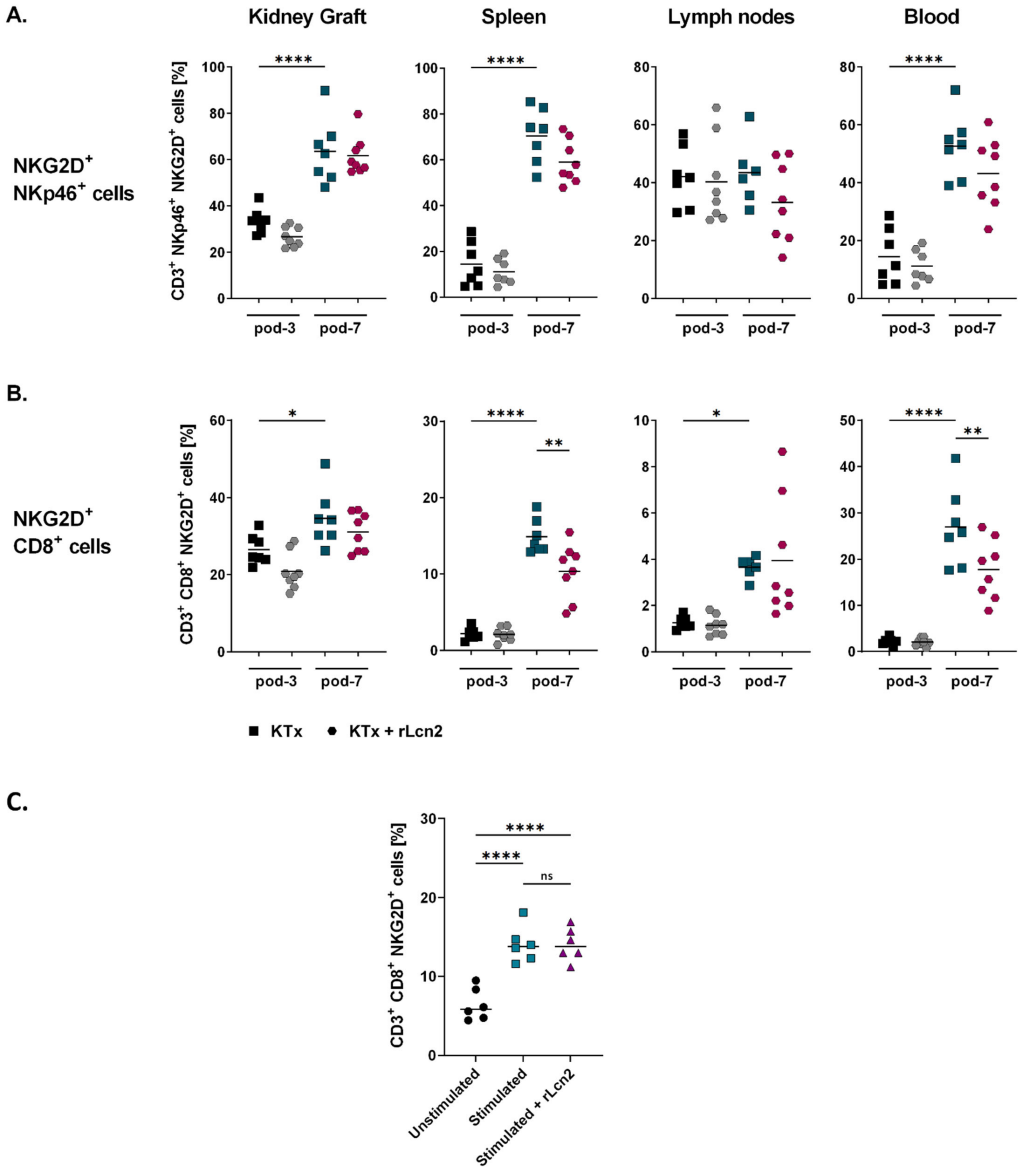
rLcn2 reduces NKG2D^+^ CD8^+^ T cells *in vivo* without direct NKG2D regulation. BALB/c to C57BL/6 kidney transplant recipients received perioperative rLcn2 (250 µg, s.c.) or vehicle. NKG2D expression on CD8^+^ T **(A)** and NK cells **(B)** was analyzed in kidney grafts, spleens, lymph nodes, and blood at pod-3 and pod-7 by flow cytometry. rLcn2 treatment did not alter NKG2D^+^ NK cells but reduced frequencies of NKG2D^+^ CD8^+^ T cells in spleen and blood at pod-7. **(C)** MACS-purified splenic T cells from naïve C57BL/6 mice were stimulated *in vitro* with ImmunoCult™ Mouse T Cell Activator for 72 hours in the presence or absence of rLcn2 and analyzed for NKG2D expression by flow cytometry. Gating strategies are shown in **Supplementary Figure S3**. Scatter plots show the relative frequencies (%) of the indicated cell types. Data are presented as mean (n = 7 KTx pod-3 and pod-7, n = 8 KTx + rLcn2 pod-3 and pod-7) **(A, B)** and (n = 6) **(C)**; statistical significance was determined by ordinary one-way ANOVA (*p < 0.05, **p ≤ 0.01, ***p ≤ 0.001, ****p ≤ 0.0001).

NKG2D is a critical activating receptor primarily responsible for detecting and eliminating transformed or infected cells. It is predominantly expressed on activated natural killer (NK) cells and is strongly induced on CD8^+^ T cells upon activation (28, 29). We observed significantly higher frequencies of NKG2D^+^ NK cells (in spleen, kidney graft and blood), and NKG2D^+^ CD8^+^ T cells (in spleen, lymph nodes, kidney graft, and blood) at pod-7 compared to pod-3 (**Figures 3A, B**). Although perioperative treatment with rLcn2 did not alter NKG2D^+^ NK cell populations (**Figure 3A**), we noted a significant reduction in the frequencies of NKG2D^+^ CD8^+^ T cells in spleen and blood of rLcn2-treated mice at pod-7 (**Figure 3B**).

To determine whether rLcn2 exerts a direct effect on CD8^+^ T cells by modulating NKG2D expression, we performed a simple *in vitro* assay in which purified pan T cells from the spleens of naïve C57BL/6 mice were activated for 72 hours in the presence and absence of rLcn2 (2 µg/mL). This experiment served as a negative validation approach to test the alternative hypothesis that rLcn2 directly regulates NKG2D expression. As expected, T cell activation markedly increased the proportion of NKG2D^+^ CD8^+^ T cells compared to unstimulated controls (**Figure 3C**). Adding rLcn2 during activation did not alter the frequency of NKG2D^+^ CD8^+^ T cells. These results indicate that rLcn2 does not directly modulate NKG2D expression on CD8^+^ T cells *in vitro*, supporting the interpretation that the *in vivo* effects of rLcn2 are likely mediated through indirect mechanisms rather than direct regulation of NKG2D.

### rLcn2 selectively affects innate immune cells in kidney transplant recipients

**Figure 4:**
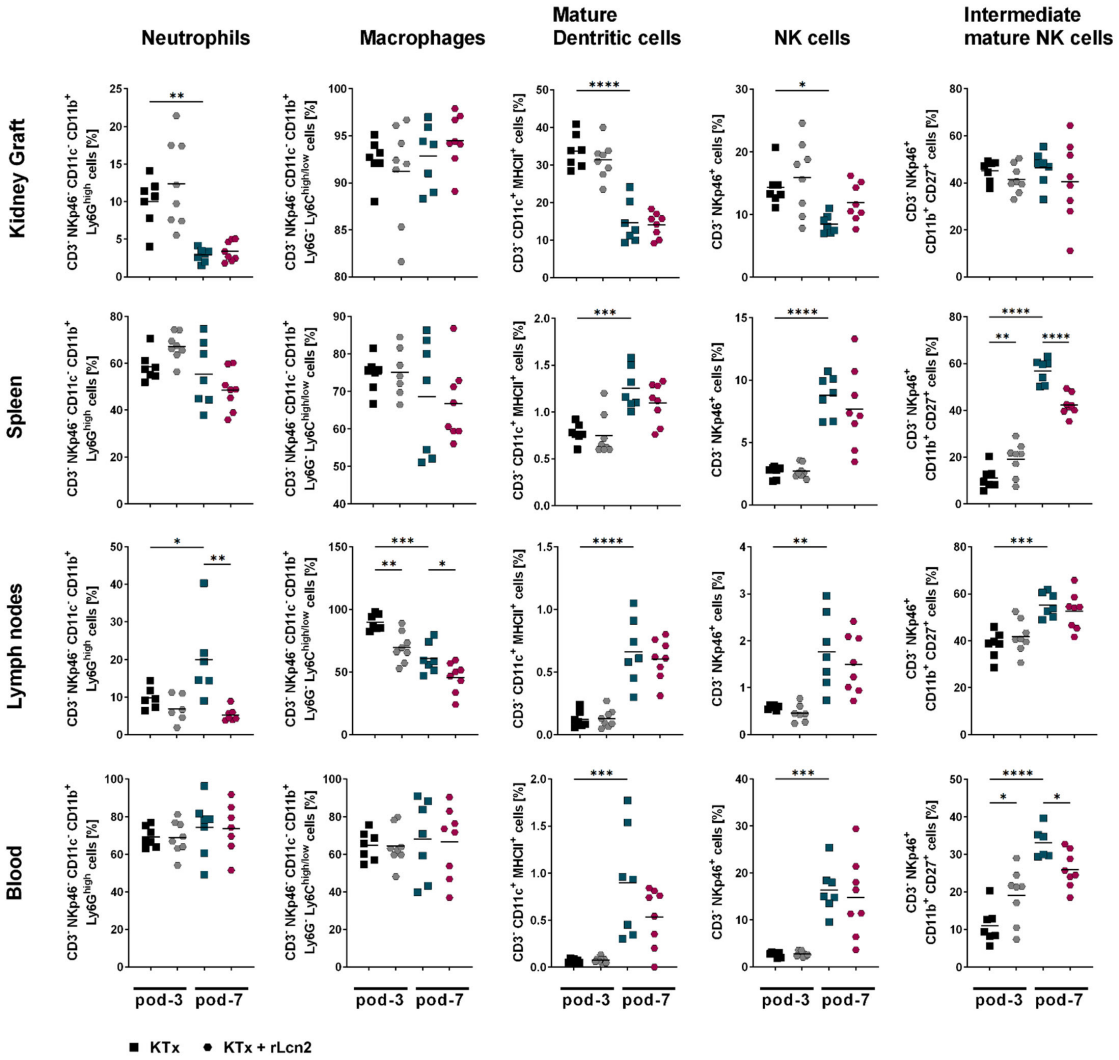
rLcn2 has minimal impact on innate immune cells post-transplant. BALB/c to C57BL/6 kidney transplant recipients received perioperative rLcn2 (250 µg, s.c.) or vehicle. Innate immune cell subsets were analyzed in kidney grafts, spleens, lymph nodes, and blood at pod-3 and pod-7 by flow cytometry. Gating strategies are described in **Supplementary Figures S3, S4**. rLcn2 had minimal impact overall but selectively reduced neutrophils and macrophages in lymph nodes and intermediate mature NK cells in spleen and blood. Scatter plots show the relative frequencies (%) of the indicated cell types. Data are presented as mean (n = 7 KTx pod-3 and pod-7, n = 8 KTx + rLcn2 pod-3 and pod-7); statistical significance was determined by ordinary one-way ANOVA (*p < 0.05, **p ≤ 0.01, ***p ≤ 0.001, ****p ≤ 0.0001).

Innate immunity plays a crucial role in the early phase of allograft rejection by detecting foreign antigens and graft released damage-associated molecular patterns (DAMPs), thereby triggering inflammatory responses. To explore whether rLcn2 confers renoprotection of the allograft by modulating the innate immune system, we performed an in-depth analysis of innate immune cell populations. Unlike T cells, the frequencies of some innate immune cells, including neutrophils, dendritic cells (DCs) and NK cells were lower in the kidney grafts at pod-7 compared to pod-3, whereas the frequencies of eosinophils, macrophages, M2 macrophages, naive and intermediate mature NK cells remained unchanged (**Figure 4**, **Supplementary Figure S5**). However, higher frequencies of mature DCs, NK cells, and intermediate mature NK cells were observed in spleens, lymph nodes, and blood at pod-7 compared to pod-3.

rLcn2 treatment did not affect the frequencies of innate immune cells in kidney grafts at either pod-3 or pod-7. Similarly, no significant differences were observed in the frequencies of innate immune cells isolated from spleens and blood, except for intermediate mature NK cells, which were more abundant at pod-3 and significantly lower at pod-7 in the rLcn2-treated animals (**Figure 4**). *Ex vivo* functional analysis of intragraft and splenic NK cells also revealed no major changes in degranulation capacity or cytokine production, except for a significant reduction in the degranulation capacity of splenic NK cells in the rLcn2-treated group (**Supplementary Figure S6**). However, compared to untreated controls, rLcn2 treatment led to significantly lower frequencies of neutrophils at pod-7 and macrophages at both pod-3 and pod-7 in the lymph nodes. Conversely, the frequencies of eosinophils in the lymph nodes were significantly increased at both pod-3 and pod-7 following rLcn2 treatment. In summary, rLcn2 appears to only have minimal effects on the innate immune system, with selective modulations of neutrophils, macrophages and intermediately mature NK cells.

### rLcn2 has minimal impact on ischemia-reperfusion injury of the mouse kidney transplant

**Figure 5:**
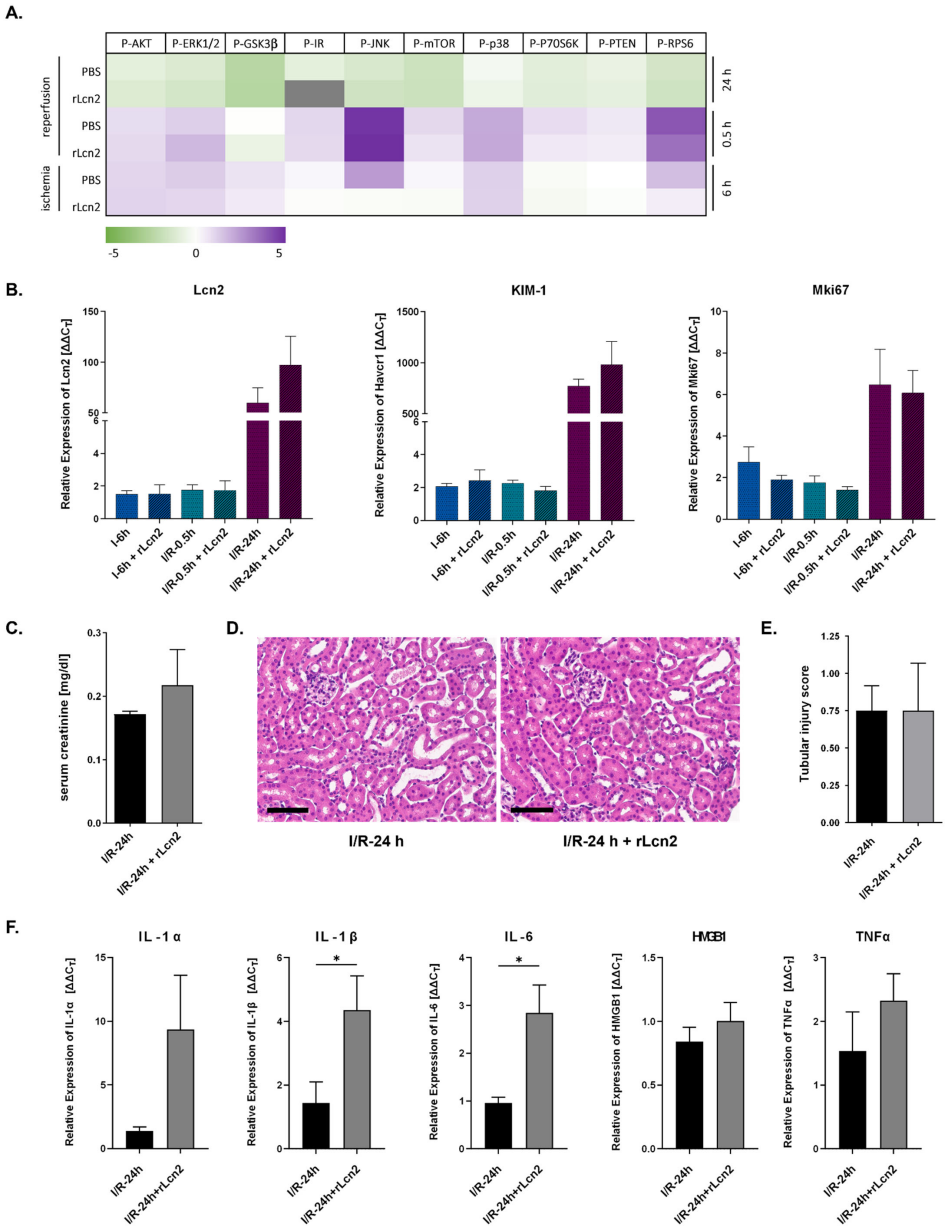
rLcn2 does not affect ischemia-reperfusion injury in syngeneic grafts. C57BL/6 to C57BL/6 syngeneic kidney grafts were subjected to 6 hours of cold ischemia followed by reperfusion for 0, 0.5 or 24 hours, with or without perioperative rLcn2 (250 µg, s.c.) treatment. **(A)** Signaling pathways (Akt/mTOR, MAPK, JAK/STAT, NFκB) were analyzed using multiplex assays covering 19 candidate molecules. Results are presented as a heatmap showing mean fluorescent intensity (MFI) of the 10 detectable phosphorylated analytes. **(B)** mRNA expression of Lcn2, KIM-1 and Mki67 in the renal isografts at 0, 0.5- and 24-hours post-transplantation. **(C)** Serum creatinine levels at 24 hours post-transplantation, comparing untreated and rLcn2-treated groups. **(D)** Representative hematoxylin and eosin-stained sections of the kidney grafts at 24 hours post-transplantation depicting similar kidney histology between untreated and rLcn2 treated animals (scale bar, 70 µm). Tubular injury score of the isografts is presented as bar graphs **(E)**. **(F)** mRNA expression of different cytokines in the renal isografts at 24 hours post-transplantation. Data are presented as mean (n = 5). Statistical significance was determined by using an unpaired t-test for two-group comparisons and by ordinary one-way ANOVA for multiple group comparisons. (*p < 0.05).

In addition to the allograft rejection driven by alloantigen-stimulated immune responses in the recipient, ischemia-reperfusion injury (IRI) causes substantial damage to the transplant during the early phase of reperfusion. We investigated whether rLcn2 could protect allografts by curtailing IRI-induced early tissue damage and inflammation and/or by promoting survival signaling. This rationale is further supported by prior work showing that rLcn2 mitigates acute kidney injury through receptor-mediated uptake by tubular epithelial cells (18, 19). In the allogeneic setting, graft injury is dominated by the adaptive alloimmune response, making it difficult to isolate and quantify the contribution of IRI. Therefore, to specifically assess the effects of rLcn2 on transplant-associated IRI, syngeneic kidney transplantations were performed (C57BL/6 to C57BL/6 mice), with kidney grafts subjected to 6 hours of cold ischemia followed by reperfusion durations of 0, 0.5 or 24 hours. Signaling analyses were conducted using multiplex assays covering 19 molecules involved in the Akt/mTOR, MAPK, JAK/STAT, and NFκB pathways (**Supplementary Table S3**). Eight molecules (CREB, NFκB, p70S6K, STAT3, STAT5, IGF1R, IRS1, and GSK3β) were below the detection limit and excluded from the analysis. While prolonged cold ischemia down-regulated the activity of most signaling molecules in the kidney grafts, their activity was markedly increased upon reperfusion (**Figure 5A**, **Supplementary Figure S7**). However, rLcn2 treatment did not result in any statistically significant changes in the activity of these signaling molecules at any of the time points examined.

The mRNA expression of kidney injury markers (Lcn2, kidney injury molecule: Kim-1) and proliferation marker (Mki67) indicated the prevalence of injury and repair responses 24 hours post-transplantation. However, these responses remained unaffected by peri-operative treatment of rLcn2 (**Figure 5B**). Similarly, functional assessments (serum creatinine levels; **Figure 5C**) and histological analyses (acute tubular necrosis) of the isografts (**Figures 5D, E**) at 24 hours post-transplantation revealed no significant differences between the untreated and rLcn2-treated groups. Analysis of proinflammatory cytokine expression at 24 hours post-transplantation revealed a significant increase in IL-1β and IL-6 in the rLcn2-treated group, whereas IL-1α, TNFα, and HMGB1 levels remained unchanged (**Figure 5F**), indicating a modest enhancement of inflammatory signaling without impacting overall tissue injury. Collectively, these findings demonstrate that rLcn2 does not influence stress, inflammatory, or survival signaling pathways, nor does it affect tissue injury or repair responses in mouse kidney grafts following transplant-associated IRI.

## Discussion

Previous research has shown that perioperative administration of rLcn2 significantly improves allograft functionality and morphology in a mouse kidney transplantation model (23). This study explores the molecular and cellular events underlying rLcn2’s renoprotective effects. Typically, a transplant faces two primary challenges from the outset: (i) the recipient’s alloimmune response due to donor-recipient incompatibility and (ii) alloimmune-independent damage caused by unavoidable ischemia-reperfusion injury (IRI). We investigated both mechanisms simultaneously to assess the course of rLcn2 mediated-renoprotection of kidney grafts using mouse models of allogeneic and syngeneic kidney transplantation. While graft injury in the allogeneic setting is primarily driven by the adaptive alloimmune response, the syngeneic model provides a system free of alloimmune activation that allows specific assessment of rLcn2’s effects on transplant-associated IRI.

To decipher the potential role of rLcn2 in modulating alloimmune responses we primarily focused on T cells, as they are the primary mediators of adaptive immune responses against the allograft, directly recognizing donor antigens and orchestrating cellular and humoral attacks that lead to graft damage (30). We observed a selective modulation of T cells, helper (T_h_) and cytotoxic (T_c_) T cells, along with their effector memory subsets (TEM, T_c_-EM, T_h_-EM), in the graft, lymphoid tissues, and blood of the rLcn2-treated mice. T_h_ cells play a central role in regulating immune responses, including allograft rejection, by differentiating into effector subsets such as T_h_1 and T_h_17, which drive specific cytokine secretion, immune cell recruitment, and cytotoxic activity (31, 32). The observed reduction in frequencies of T_h_ cells in the kidney graft and lymph nodes of the rLcn2-treated mice suggests an immunomodulatory role of rLcn2, likely mitigating graft inflammation and lowering the risk of rejection.

T_c_ cells are crucial mediators of allograft rejection, excreting direct cytotoxicity through the release of molecules such as perforin and granzyme. Although overall T_c_ cell infiltration into the graft was comparable between rLcn2-treated and untreated groups, T_c_ cells isolated from the grafts of the treated group exhibited markedly reduced degranulation, as well as diminished production of IFNγ and perforin. This indicates that rLcn2 treatment impairs T_c_ cell cytotoxic function without affecting their recruitment, thereby contributing to graft protection.

Effector memory T cells contribute to allograft rejection by rapidly responding to donor antigens without the need for activation in lymph nodes. These pre-activated T cells infiltrate the transplanted organ and quickly release inflammatory cytokines, thereby amplifying tissue injury and promoting inflammation (33). The observed selective reduction in TEM, T_c_-EM, T_h_-EM with rLcn2 treatment suggests a potential dampening of immune memory, indicating that rLcn2 may help modulate long-term immune responses. Recent studies highlight that TEM cells, due to their resistance to conventional immunosuppressive therapies and capacity to sustain inflammation, are key drivers in the progression of chronic rejection (34).

rLcn2 appears to have a minimal effect on the innate immune system, with selective modulations of neutrophils, macrophages in lymph nodes. The accumulation of macrophages and neutrophils in the draining lymph nodes may reflect preparatory steps toward an adaptive immune response (35–37). While this does not necessarily indicate ongoing allograft rejection, it may precede it. The targeting of these innate immune cells within lymph nodes suggests a potential role of rLcn2 in modulating early events of the alloimmune response occurring in secondary lymphoid tissues.

NK cells provide rapid responses against infected or transformed cells and can directly kill target cells without prior sensitization (38). We recently demonstrated that NK cells evade targeting by calcineurin inhibitors (CNIs), resulting in incomplete protection of the immunosuppression from allograft rejection. Moreover, NK cell depletion combined with the CNI cyclosporin A synergistically improved graft function and prolonged graft survival (25). However, here we found that while the treatment with rLcn2 did not affect the pan NK cells, the frequencies of a subset, i.e. intermediate mature NK cells, were reduced in blood and spleen. Intermediate mature NK cells are known for a high cytokine release and killing capacity (39, 40). The decrease in intermediate mature NK cells suggests a potential modulation of NK cell activity, which can prevent excessive cytotoxicity that may lead to graft rejection.

NKG2D is a key activating receptor expressed on NK cells and CD8^+^ T cells. It recognizes stress-induced ligands on target cells, triggering NK cell activation and enhancing CD8^+^ T cell cytotoxicity by providing co-stimulatory signals (41, 42). The increased abundance of NKG2D-expressing NK and T cells at pod-7 compared to pod-3 highlights their functional relevance in the context of allograft rejection. While rLcn2 treatment did not affect the number of NKG2D^+^ NK cells, it significantly reduced frequencies of NKG2D^+^ CD8^+^ T cells in spleens and blood at pod-7, suggesting a modulatory effect of rLcn2 on cytotoxic T cells. *In vitro*, rLcn2 did not directly modulate NKG2D expression on CD8^+^ T cells, indicating that its *in vivo* effects are context dependent and mediated by the inflammatory microenvironment rather than by direct T cell regulation. Moreover, because NKG2D expression on CD8^+^ T cells is generally induced upon T cell activation within an inflammatory milieu (28, 43), its modulation *in vivo* may reflect the overall immune response rather than a direct target of rLcn2.

Lcn2 demonstrates context-dependent, dual roles in inflammation and immune regulation. On one hand, it acts as an innate immune acute-phase protein: it sequesters iron by binding bacterial siderophores, restricting microbial growth, and serves as a chemoattractant that promotes neutrophil recruitment, enhances cytokine production, and can amplify local inflammatory responses (9, 10). On the other hand, growing evidence indicates a protective and immunoregulatory role: Lcn2 can modulate macrophage polarization toward anti-inflammatory phenotypes, can induce CD8^+^ T cell apoptosis and support tissue repair and regeneration following injury (44–46). In the context of allogeneic transplant model, the beneficial effects observed after rLcn2 administration likely reflect the immunoregulatory action of Lcn2.

Importantly, the lack of any observable effect on innate immune cells within the kidney graft itself indicates that rLcn2 does not directly regulate intragraft inflammation or injury. This observation aligns with our analyses of inflammatory, survival, and stress signaling pathways, as well as functional and histological assessments in syngeneically transplanted mouse kidneys subjected to prolonged cold ischemia (6 hours), all of which showed no significant impact of rLcn2 on IRI-induced damage. In contrast, other studies have reported a protective effect of rLcn2 in models of acute kidney injury (AKI) following short durations of warm ischemia (30 minutes) (18, 19). This protection is attributed to the receptor-mediated uptake of the rLcn2:Siderophore: Fe complex by proximal tubular cells, enabling intracellular delivery of iron (19). Iron may confer protection during IRI through its essential roles in oxygen transport, mitochondrial function, and antioxidant defense (47–49). Excessive iron, however, can cause cell death and tissue damage, primarily by promoting the production of reactive oxygen species (ROS) (50). It remains unclear whether endogenously produced Lcn2 is iron-loaded and capable of this delivery. Also, endogenous Lcn2 induction may occur too late to effectively prevent damage.

We propose that the lack of rLcn2-mediated protection from transplant-associated IRI in our study stems from the different nature of ischemia, as our kidney transplant model employs cold ischemia in contrast to the warm ischemia used in earlier investigations. In our setting, the grafts exhibited low-grade tubular injury without notable functional deterioration at 24 hours post-transplantation, suggesting that the extent of cold ischemic injury may have been too mild to reveal a clear therapeutic benefit of rLcn2. Notably, while no clear vascular lesions were observed in the mouse kidney transplants, our recent work demonstrated that sGC activator cinaciguat-mediated dilation of afferent arterioles isolated from the mouse kidney grafts was impaired due to prolonged cold ischemia (26). Pretreatment with rLcn2 preserved this dilation response, suggesting a protective effect of rLcn2 through modulation of sGC-mediated afferent arteriolar tone (26). In contrast, at the same time point, i.e. 24 hours following AKI induced by warm ischemia, pronounced tubular necrosis and significant functional impairment have been observed (18, 19). In warm ischemia, the kidney continues to have high metabolic demands despite lack of oxygen, leading to rapid ATP depletion, oxidative stress, and acute tubular necrosis. This triggers immediate and severe injury involving both necrosis and apoptosis, along with a strong inflammatory response (51, 52). Conversely, cold ischemia, as used during organ preservation, slows down metabolism and delays the onset of cellular injury. Nevertheless, damage can still occur upon reperfusion, with oxidative stress and immune activation contributing to delayed graft function (53, 54).

We recognize a few limitations in our study. First, most of our flow cytometry analyses relied on relative frequencies. Although this approach effectively captures shifts in immune composition, it may not fully reflect differences in absolute cell numbers between groups. Nonetheless, relative frequencies are less sensitive to technical variability in tissue digestion and cell yield, making them a robust metric for comparing proportional changes across experimental groups and therefore suitable for this study. Second, our analyses in the allogeneic mouse kidney transplantation model were focused on the early (pod-3) and effector (pod-7) phases of alloimmune injury, leaving earlier (pod-1-2) and later (pod-10-14) dynamics unexamined. Future studies incorporating these time points will provide a more complete understanding of how rLcn2 shapes graft immune responses over time.

## Conclusion

This study shows that perioperative rLcn2 therapy selectively affects the immune system by mainly suppressing adaptive immunological responses in a mouse kidney transplant model. With minimal impact on innate immune cells or pathways implicated in tissue inflammation and repair, rLcn2 significantly reduces T cell activation and cytotoxicity.

## Data Availability

The raw data supporting the conclusions of this article will be made available by the authors, without undue reservation.
