# Supplementary figures and images for: Lipocalin-2 modulates recipients alloimmune responses to the murine kidney transplants
Source: Front Immunol. 2025 Dec 19;16:1716393. doi: 10.3389/fimmu.2025.1716393 (PMC12757278; doi:10.3389/fimmu.2025.1716393)

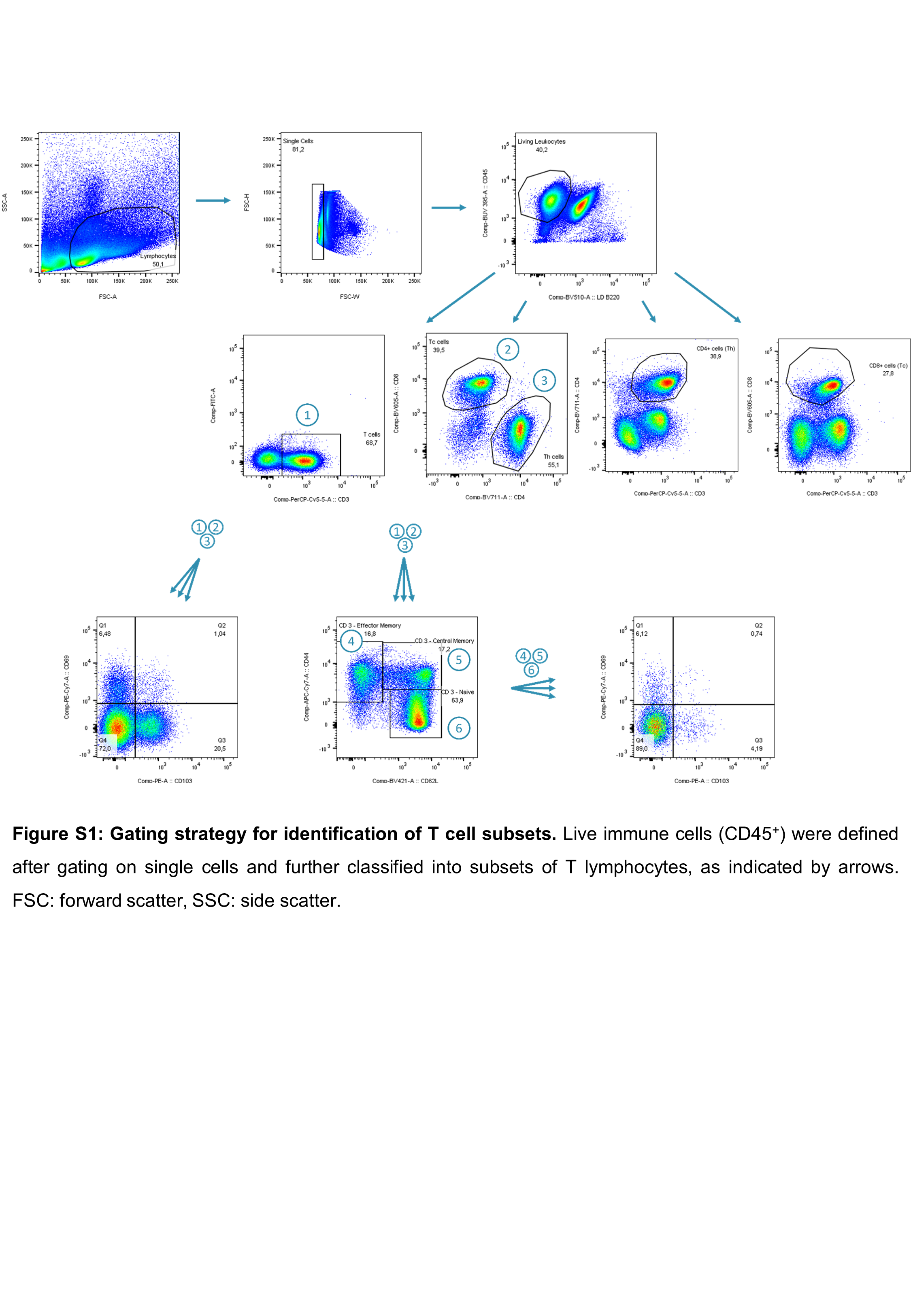

Supplement: Supplementary file 1 [file Image1.tif]

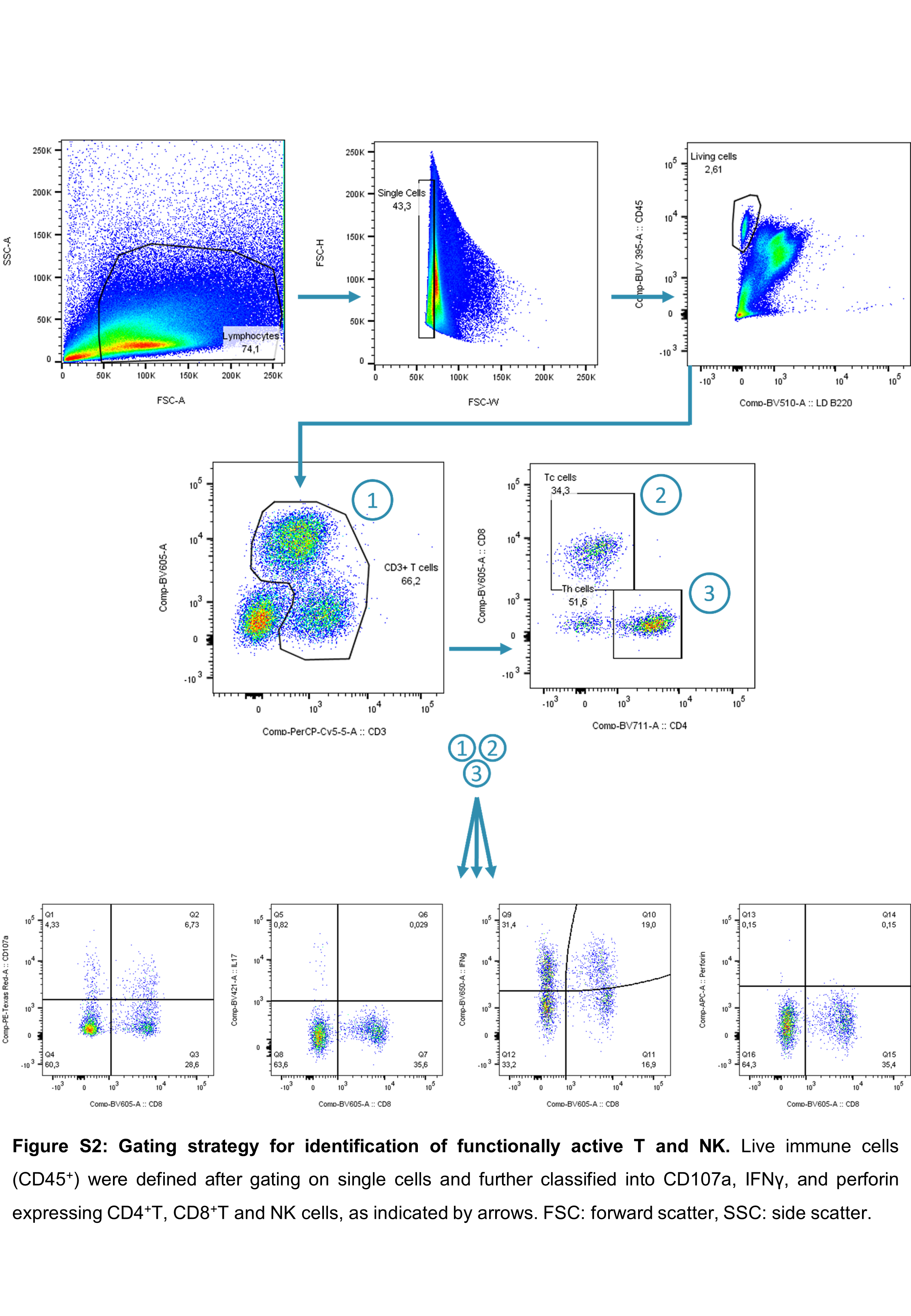

Supplement: Supplementary file 2 [file Image2.tif]

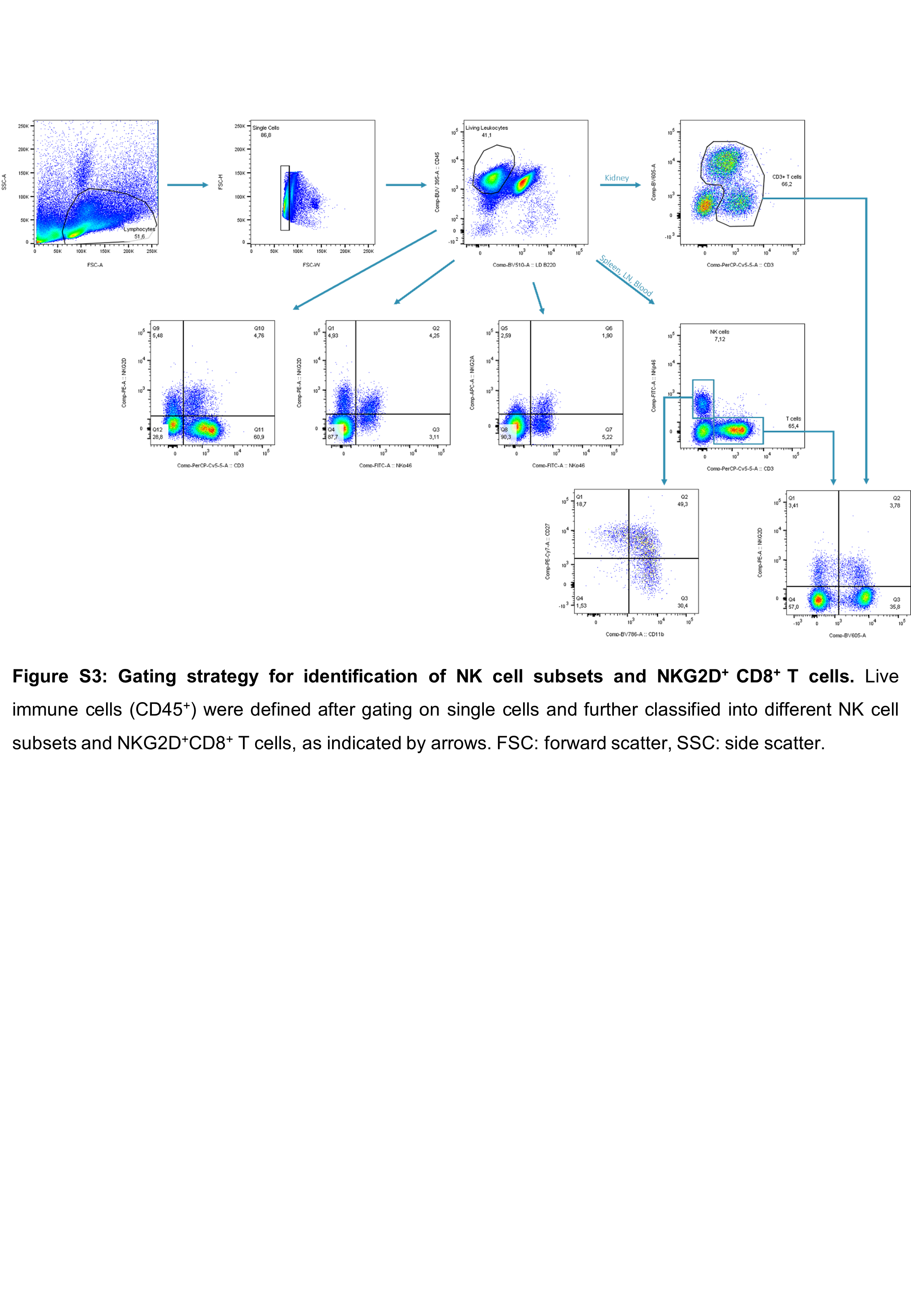

Supplement: Supplementary file 3 [file Image3.tif]

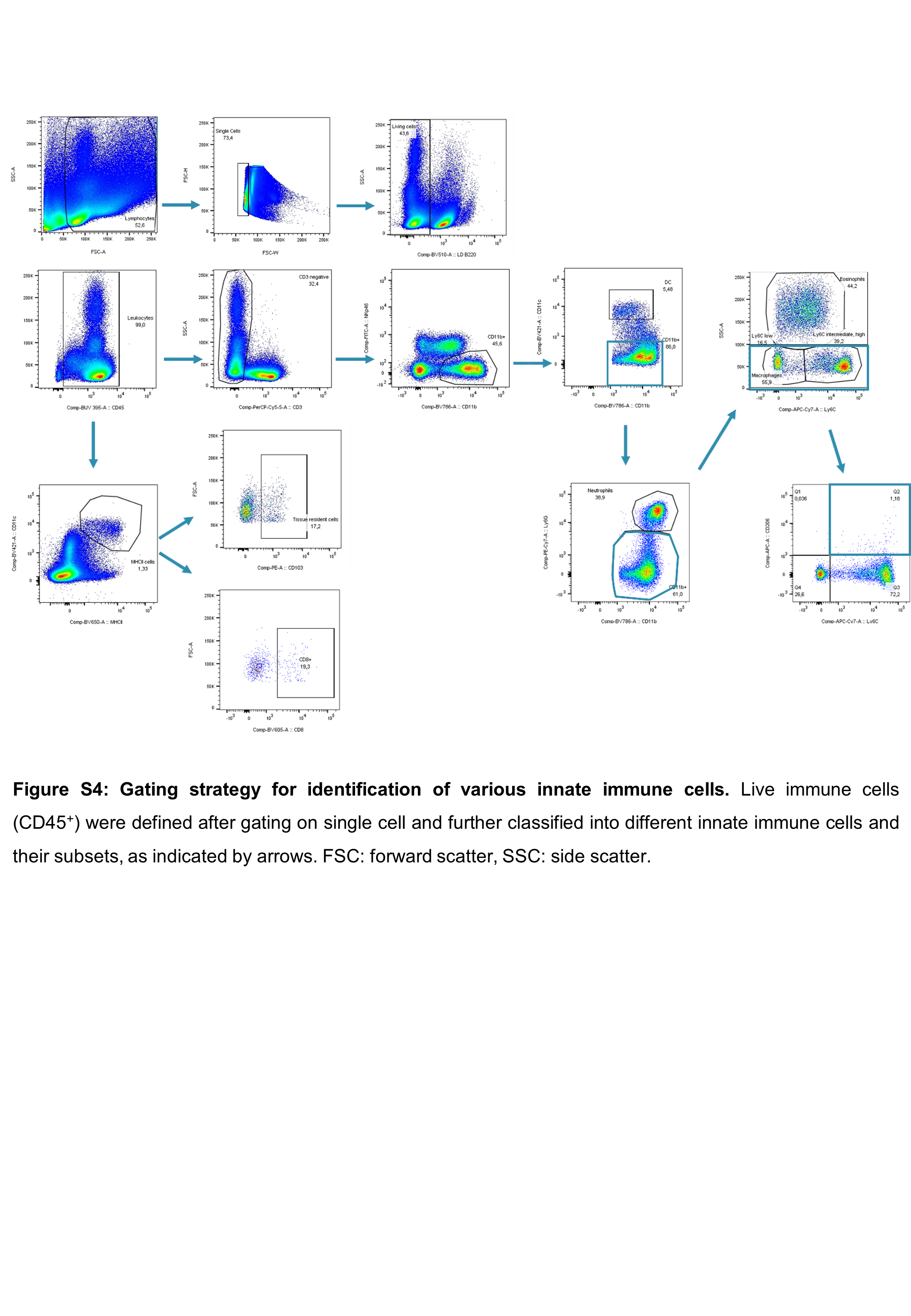

Supplement: Supplementary file 4 [file Image4.tif]

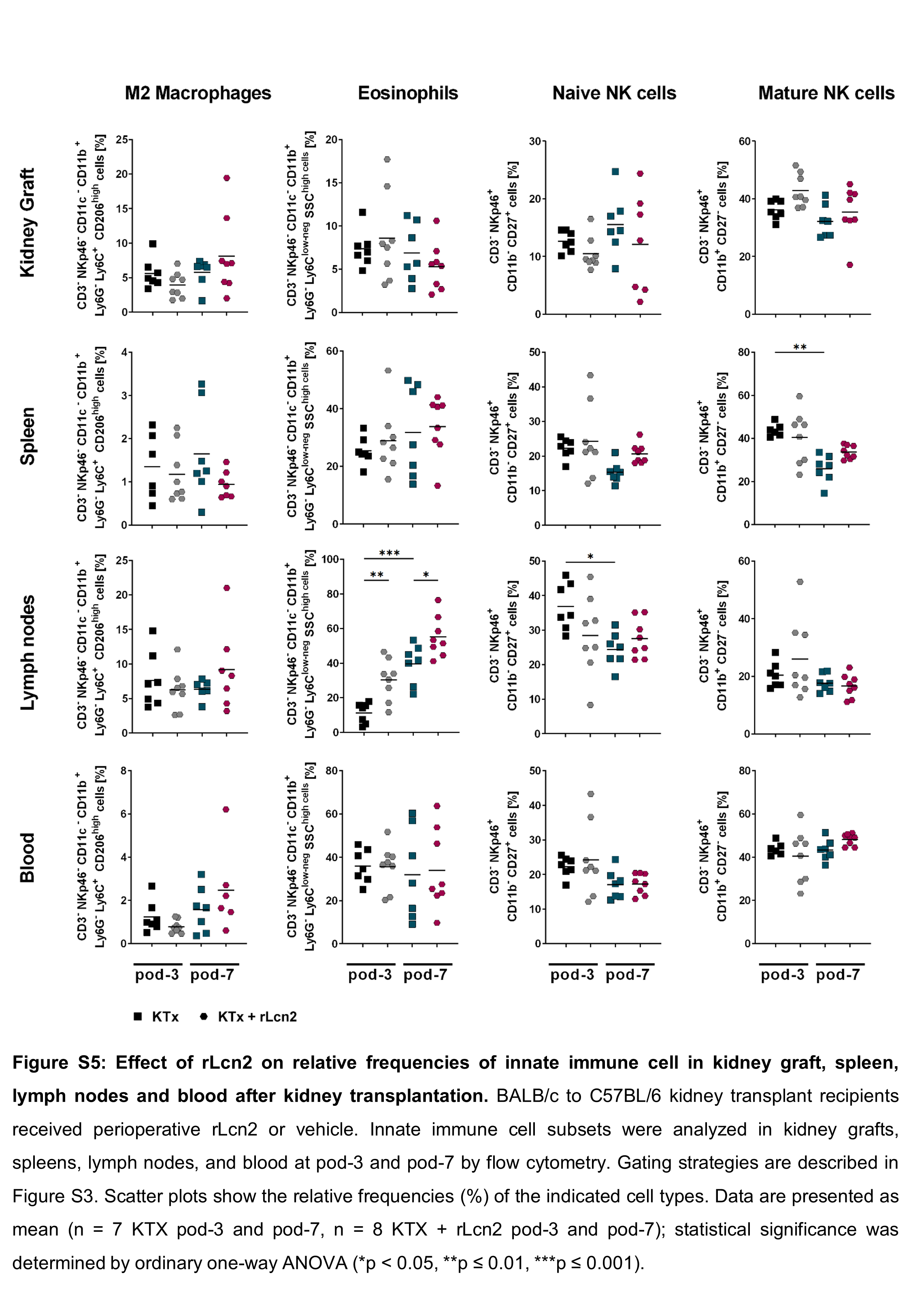

Supplement: Supplementary file 5 [file Image5.tif]

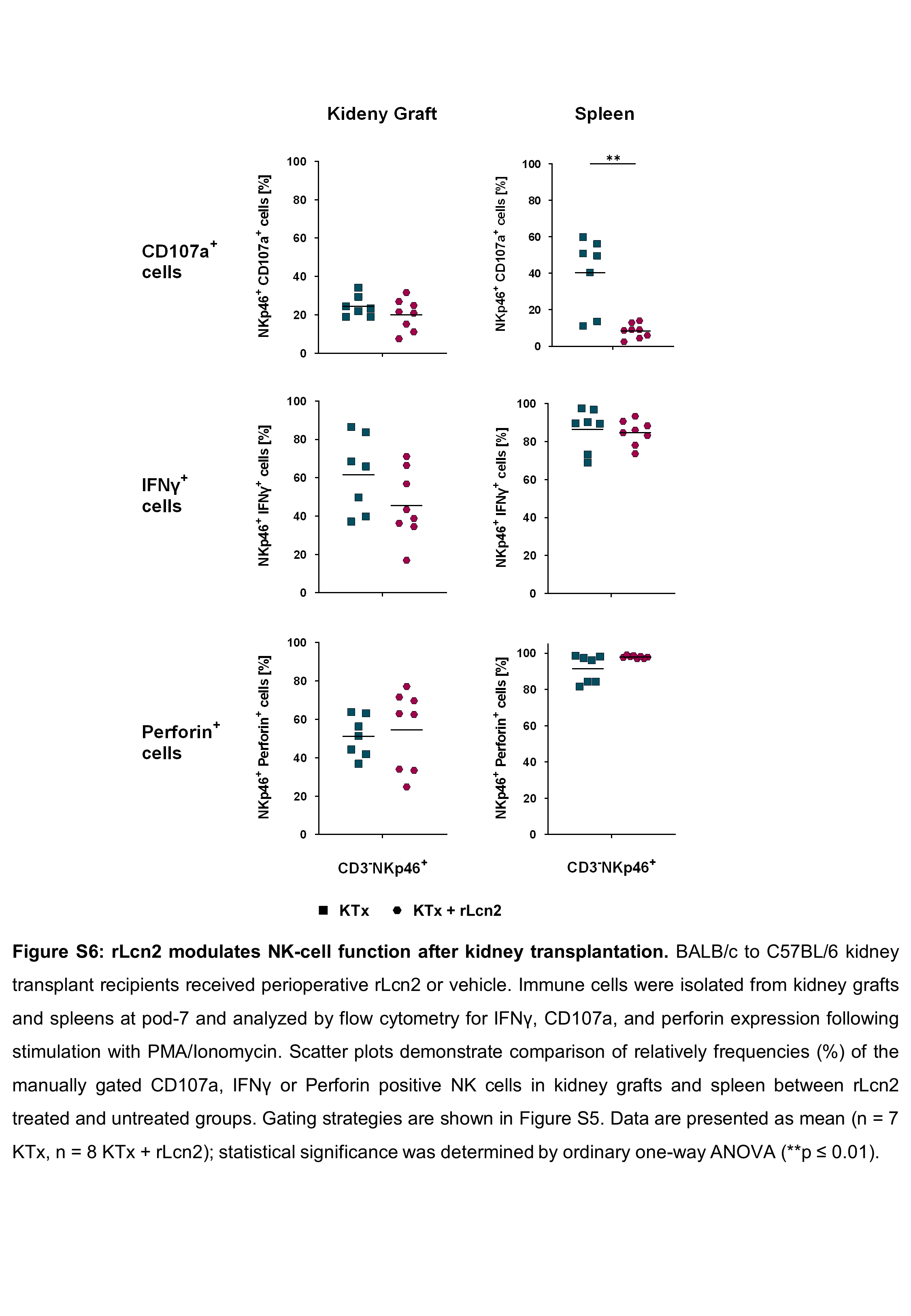

Supplement: Supplementary file 6 [file Image6.tif]

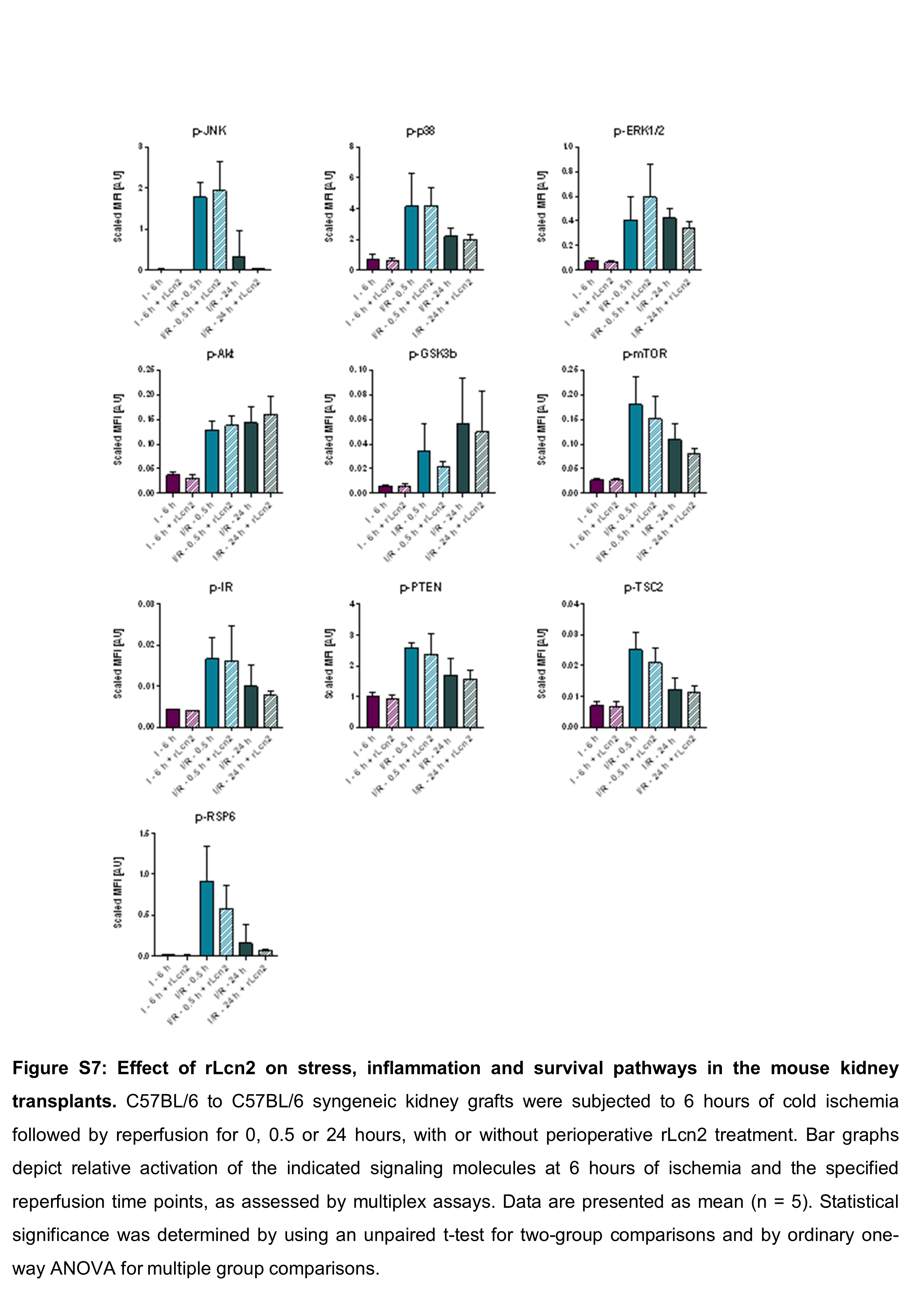

Supplement: Supplementary file 7 [file Image7.tif]
