# Supplementary material for: Lipocalin-2 modulates recipients alloimmune responses to the murine kidney transplants
Source: Front Immunol. 2025 Dec 19;16:1716393. doi: 10.3389/fimmu.2025.1716393 (PMC12757278; doi:10.3389/fimmu.2025.1716393)
Supplement: Supplementary file 8 [file Table1.docx]

**Table S1: Panels used for staining of isolated cells from spleen, lymph nodes, kidney, and blood.**

| **Marker** | **Clones** | | **Fluorochrome** | **Manufacturer** | **Dilutions** |  | **Marker** | **Clones** | | **Fluorochrome** | **Manufacturer** | **Dilutions** |
| --- | --- | --- | --- | --- | --- | --- | --- | --- | --- | --- | --- | --- |
|  | ***NK-Panel*** | |  |  |  |  |  | ***T-Panel*** | |  |  |  |
| NKp46 | 29A1.4 | | FITC | Biolegend | 1: 100 |  | CD103 | 2E7 | | PE | Biolegend | 1: 200 |
| CD27 | LG.3A10 | | PE-Cy7 | Biolegend | 1: 100 |  | CD69 | H1.2F3 | | PE-Cy7 | Biolegend | 1: 200 |
| CD3 | REA641 | | PerCP-Vio700 | Miltenyi | 1: 400 |  | CD3 | REA641 | | PerCP-Vio700 | Miltenyi | 1: 400 |
| CD45 | 30-F11 | | UV395 | BD | 1: 400 |  | CD45 | 30-F11 | | UV395 | BD | 1: 400 |
| B220 | RA3-6B2 | | BV510 | Biolegend | 1: 400 |  | B220 | RA3-6B2 | | BV510 | Biolegend | 1: 400 |
| CD8a | 53-6.7 | | BV605 | Biolegend | 1: 800 |  | CD8a | 53-6.7 | | BV605 | Biolegend | 1: 800 |
| NKG2D | CX5 | | PE | Biolegend | 1: 800 |  | CD44 | IM7 | | APC-eFluor 780 | Thermo Fisher | 1: 800 |
| L/D |  | | BV510 | Biolegend | 1: 1000 |  | L/D |  | | BV510 | Biolegend | 1: 1000 |
| NKG2A | 16A11 | | APC | Biolegend | 1: 3200 |  | CD4 | RM4-5 | | BV711 | Biolegend | 1: 1600 |
| CD11b | M1/70 | | BV786 | Biolegend | 1: 3200 |  | CD62L | MEL-14 | | BV421 | Biolegend | 1: 3200 |
|  |  | |  |  |  |  |  |  | |  |  |  |
|  | | ***Innate-Panel*** | |  |  |  |  | | ***Functional-Panel*** | |  |  |
| NKp46 | 29A1.4 | | FITC | Biolegend | 1: 100 |  | CD107a |  | | PE-Texas-Red |  | 1: 800 |
| CD11c | N418 | | BV421 | Biolegend | 1: 200 |  |  | | *Surface Marker* | |  |  |
| CD103 | 2E7 | | PE | Biolegend | 1: 200 |  | NKp46 | 29A1.4 | | FITC | Biolegend | 1: 100 |
| CD3 | REA641 | | PerCP-Vio700 | Miltenyi | 1: 400 |  | CD3 | REA641 | | PerCP-Vio700 | Miltenyi | 1: 400 |
| CD206 | C068C2 | | AlexaFluor647 | Biolegend | 1: 400 |  | CD45 | 30-F11 | | UV395 | BD | 1: 400 |
| Ly6C | HK1.4 | | APC-Cy7 | Biolegend | 1: 400 |  | B220 | RA3-6B2 | | BV510 | Biolegend | 1: 400 |
| Ly6G | 1A8 | | PE-Cy7 | Biolegend | 1: 400 |  | CD8a | 53-6.7 | | BV605 | Biolegend | 1: 800 |
| CD45 | 30-F11 | | UV395 | BD | 1: 400 |  | L/D |  | | BV510 | Biolegend | 1: 1000 |
| B220 | RA3-6B2 | | BV510 | Biolegend | 1: 400 |  | CD4 | RM4-5 | | BV711 | Biolegend | 1: 1600 |
| CD8a | 53-6.7 | | BV605 | Biolegend | 1: 800 |  |  | | *Intracellular Marker* | |  |  |
| L/D |  | | BV510 | Biolegend | 1: 1000 |  | IL-17 | TC11-18H10.1 | | BV421 | Biolegend | 1: 100 |
| CD11b | M1/70 | | BV786 | Biolegend | 1: 3200 |  | IFNγ | XMG1.2 | | BV650 | Biolegend | 1: 200 |
| HCII | M5/114.15.2 | | BV650 | Biolegend | 1: 6400 |  | Perforin | S16009B | | APC | Biolegend | 1: 800 |
