# Supplementary material for: Lipocalin-2 modulates recipients alloimmune responses to the murine kidney transplants
Source: Front Immunol. 2025 Dec 19;16:1716393. doi: 10.3389/fimmu.2025.1716393 (PMC12757278; doi:10.3389/fimmu.2025.1716393)
Supplement: Supplementary file 9 [file Table2.docx]

Table S2: List of expression assays used for RT-qPCR analyses.

| **Expression Assay** | **Supplier** |
| --- | --- |
| HAVcr1 mouse expression assay, Mm00506686_m1 | Thermo Fisher Scientific Inc., Waltham, Massachusetts, USA |
| Lcn2 mouse expression assay, Mm01324470_m1 | Thermo Fisher Scientific Inc., Waltham, Massachusetts, USA |
| IL-1α mouse expression assay, Mm00439620_m1 | Thermo Fisher Scientific Inc., Waltham, Massachusetts, USA |
| IL-1β mouse expression assay, Mm00434228_m1 | Thermo Fisher Scientific Inc., Waltham, Massachusetts, USA |
| IL-6 mouse expression assay, Mm00446190_m1 | Thermo Fisher Scientific Inc., Waltham, Massachusetts, USA |
| HMGB1 mouse expression assay, Mm00849805_gH | Thermo Fisher Scientific Inc., Waltham, Massachusetts, USA |
| TNFα mouse expression assay, Mm00443258_m1 | Thermo Fisher Scientific Inc., Waltham, Massachusetts, USA |
| Ppia mouse expression assay, Mm02342429_g1 | Thermo Fisher Scientific Inc., Waltham, Massachusetts, USA |
