# Supplementary material for: Lipocalin-2 modulates recipients alloimmune responses to the murine kidney transplants
Source: Front Immunol. 2025 Dec 19;16:1716393. doi: 10.3389/fimmu.2025.1716393 (PMC12757278; doi:10.3389/fimmu.2025.1716393)
Supplement: Supplementary file 10 [file Table3.docx]

Table S3: List of analytes included in the multiplex analysis. Multiplex assays were performed using the Milliplex MAP 9-Plex Multi-Pathway and 11-Plex Akt/mTOR signaling kits, which included candidate analytes of stress, inflammation and survival signaling pathways. Signals for CREB, NFκB, p70S6K, STAT3, STAT5, IGF1R, IRS1, and GSK3β were below the background levels and were therefore excluded from the analysis.

|  | **Analytes** |  |
| --- | --- | --- |
|  | ***11Plex*** | ***9Plex*** |
|  | GSK3b (Ser9) | CREB |
|  | IGF1R (Tyr1135_1136) | JNK |
|  | IRS1 (Ser636) | NFκB |
|  | Akt (Ser473) | p38 |
|  | mTOR (Ser2448) | ERK1/2 |
|  | P70S6K (Thr412) | AKT |
|  | IR (Tyr1162_1163) | p70S6K |
|  | PTEN (Ser380) | STAT3 |
|  | GSK3a (Ser21) | STAT5 |
|  | TSC2 (Ser939) | beta-Tubulin |
|  | RPS6 (Ser235_236) |  |
|  | GSK3b (Ser9) |  |
|  | TSC2 (Ser939) |  |
|  | RPS6 (Ser235_236) |  |
